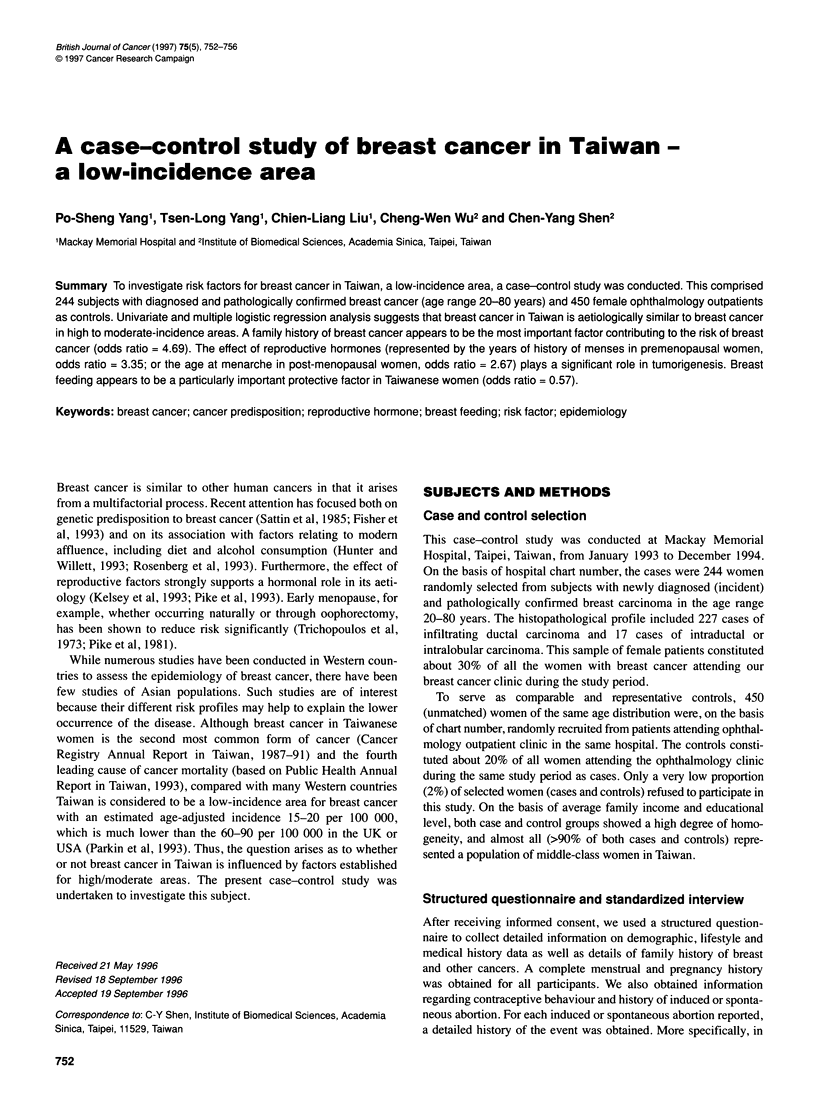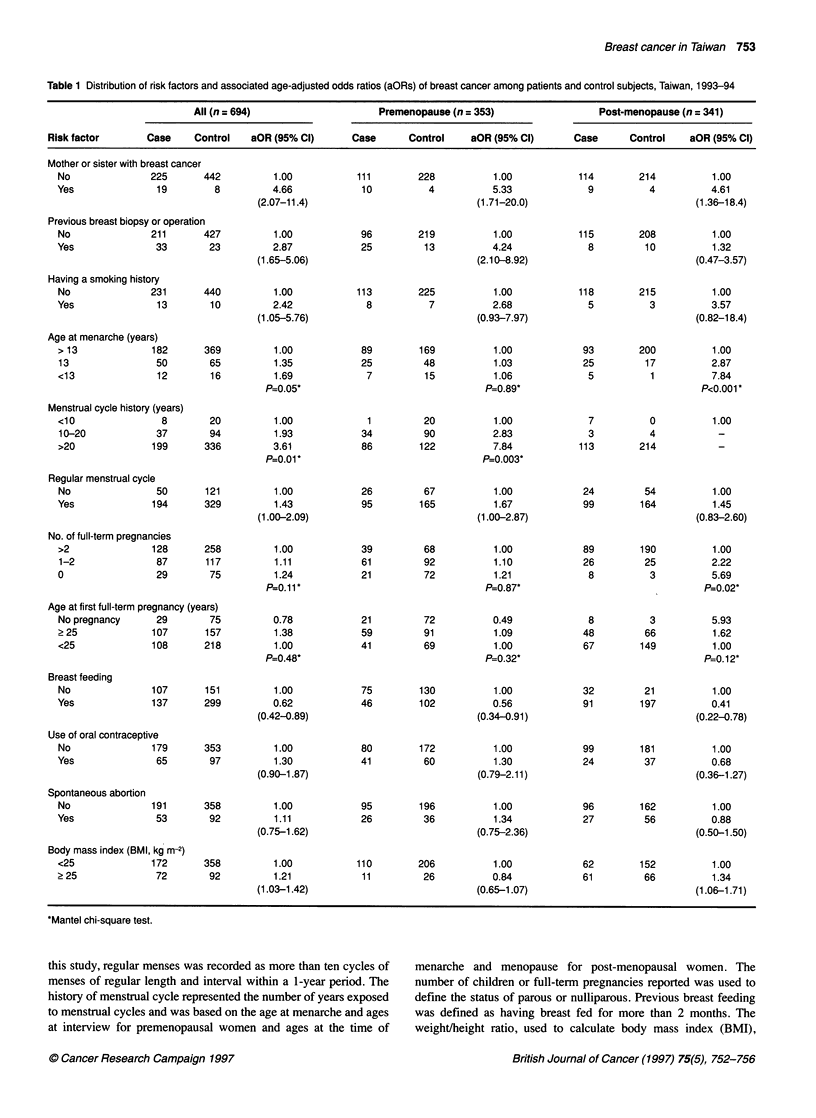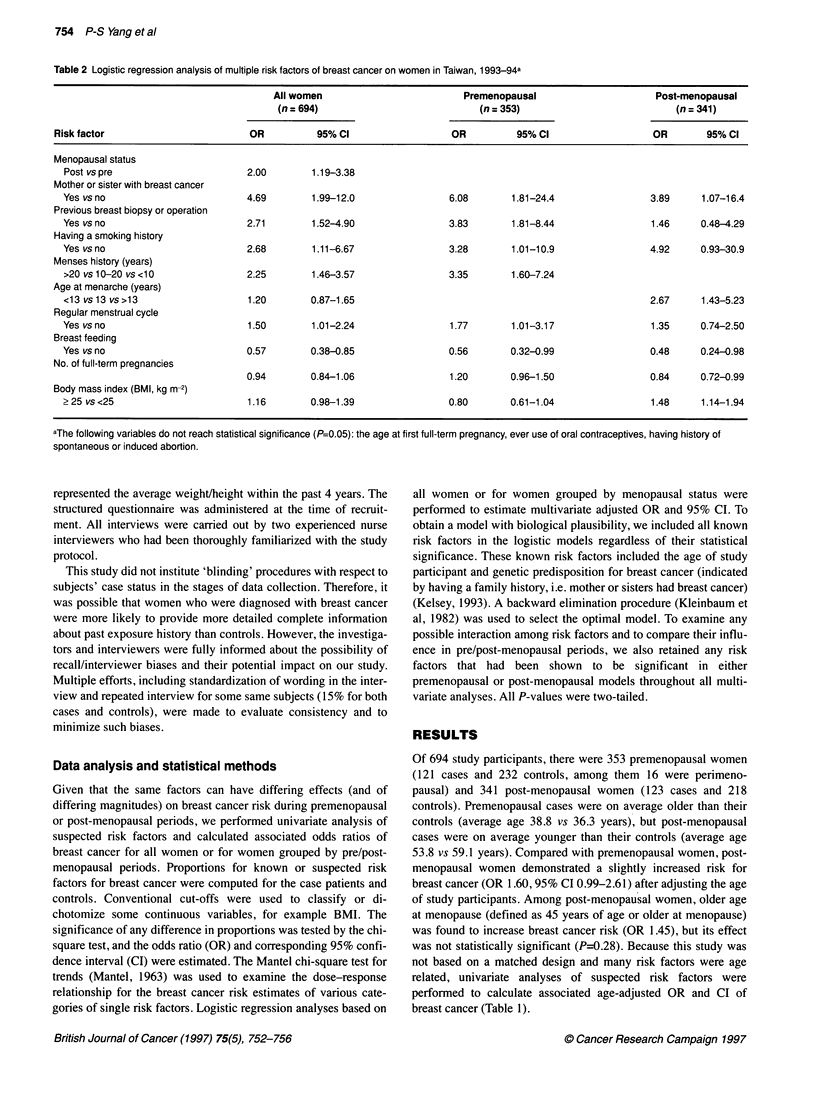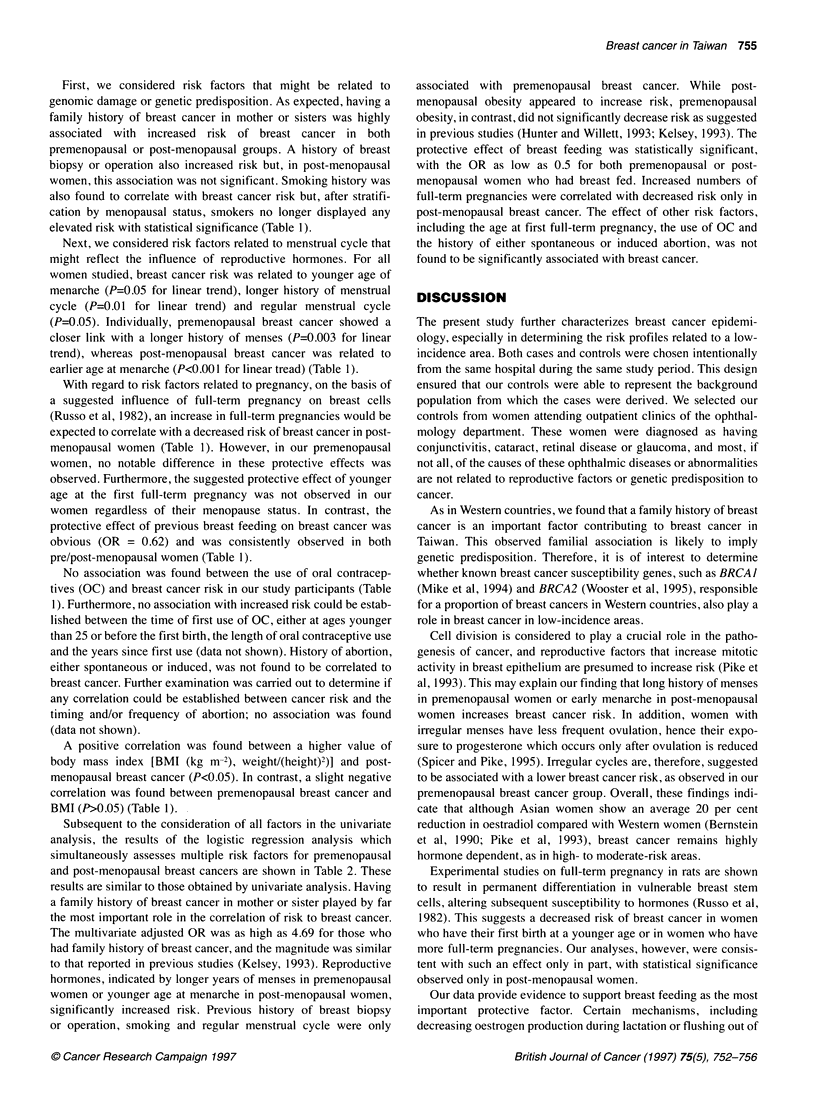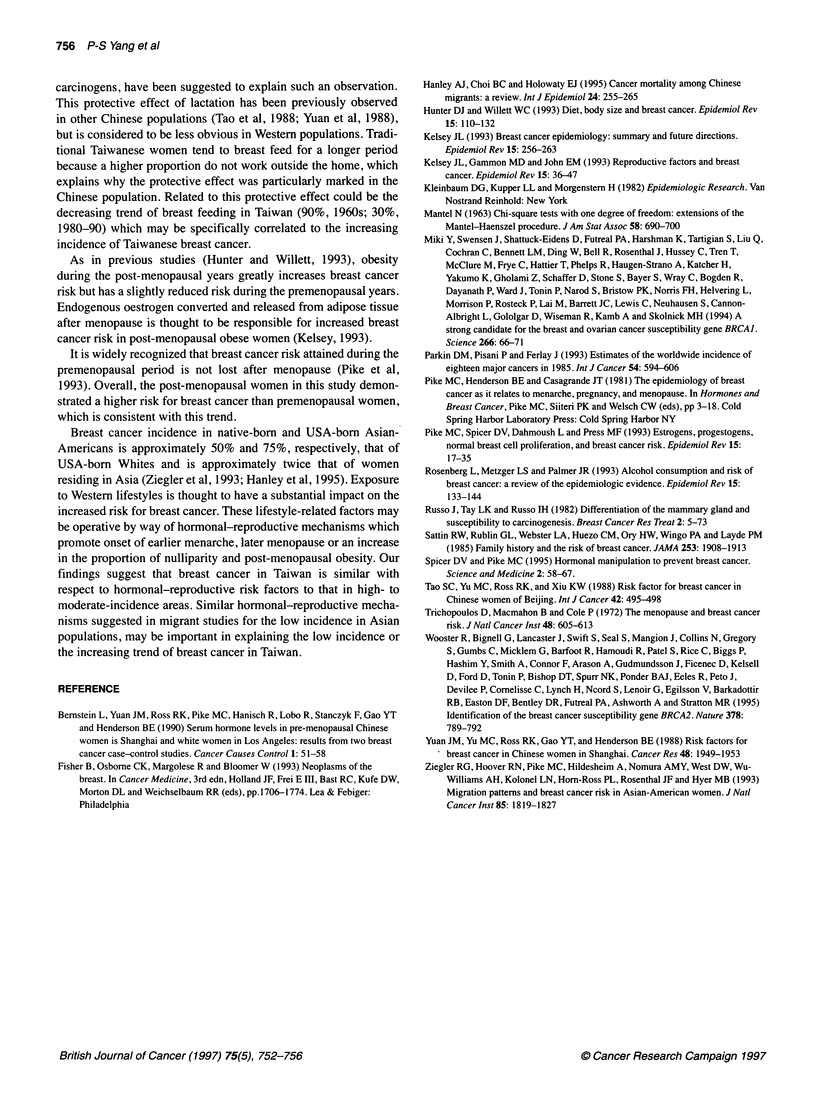# A case-control study of breast cancer in Taiwan--a low-incidence area.

**DOI:** 10.1038/bjc.1997.133

**Published:** 1997

**Authors:** P. S. Yang, T. L. Yang, C. L. Liu, C. W. Wu, C. Y. Shen

**Affiliations:** Mackay Memorial Hospital, Academia Sinica, Taipei, Taiwan.

## Abstract

To investigate risk factors for breast cancer in Taiwan, a low-incidence area, a case-control study was conducted. This comprised 244 subjects with diagnosed and pathologically confirmed breast cancer (age range 20-80 years) and 450 female ophthalmology outpatients as controls. Univariate and multiple logistic regression analysis suggests that breast cancer in Taiwan is aetiologically similar to breast cancer in high to moderate-incidence areas. A family history of breast cancer appears to be the most important factor contributing to the risk of breast cancer (odds ratio = 4.69). The effect of reproductive hormones (represented by the years of history of menses in premenopausal women, odds ratio = 3.35; or the age at menarche in post-menopausal women, odds ratio = 2.67) plays a significant role in tumorigenesis. Breast feeding appears to be a particularly important protective factor in Taiwanese women (odds ratio = 0.57).


British Journal of Cancer (1997) 75(5), 752–756
© 1997 Cancer Research Campaign

### A case–control study of breast cancer in Taiwan – a low-incidence area

**Po-Sheng Yang[1], Tsen-Long Yang[1], Chien-Liang Liu[1], Cheng-Wen Wu[2] and Chen-Yang Shen[2]**

[1]Mackay Memorial Hospital and [2]Institute of Biomedical Sciences, Academia Sinica, Taipei, Taiwan

**Summary** To investigate risk factors for breast cancer in Taiwan, a low-incidence area, a case–control study was conducted. This comprised 244 subjects with diagnosed and pathologically confirmed breast cancer (age range 20–80 years) and 450 female ophthalmology outpatients as controls. Univariate and multiple logistic regression analysis suggests that breast cancer in Taiwan is aetiologically similar to breast cancer in high to moderate-incidence areas. A family history of breast cancer appears to be the most important factor contributing to the risk of breast cancer (odds ratio = 4.69). The effect of reproductive hormones (represented by the years of history of menses in premenopausal women, odds ratio = 3.35; or the age at menarche in post-menopausal women, odds ratio = 2.67) plays a significant role in tumorigenesis. Breast feeding appears to be a particularly important protective factor in Taiwanese women (odds ratio = 0.57).

**Keywords:** breast cancer; cancer predisposition; reproductive hormone; breast feeding; risk factor; epidemiology

Breast cancer is similar to other human cancers in that it arises from a multifactorial process. Recent attention has focused both on genetic predisposition to breast cancer (Sattin et al, 1985; Fisher et al, 1993) and on its association with factors relating to modern affluence, including diet and alcohol consumption (Hunter and Willett, 1993; Rosenberg et al, 1993). Furthermore, the effect of reproductive factors strongly supports a hormonal role in its aetiology (Kelsey et al, 1993; Pike et al, 1993). Early menopause, for example, whether occurring naturally or through oophorectomy, has been shown to reduce risk significantly (Trichopoulos et al, 1973; Pike et al, 1981).

While numerous studies have been conducted in Western countries to assess the epidemiology of breast cancer, there have been few studies of Asian populations. Such studies are of interest because their different risk profiles may help to explain the lower occurrence of the disease. Although breast cancer in Taiwanese women is the second most common form of cancer (Cancer Registry Annual Report in Taiwan, 1987–91) and the fourth leading cause of cancer mortality (based on Public Health Annual Report in Taiwan, 1993), compared with many Western countries Taiwan is considered to be a low-incidence area for breast cancer with an estimated age-adjusted incidence 15–20 per 100 000, which is much lower than the 60–90 per 100 000 in the UK or USA (Parkin et al, 1993). Thus, the question arises as to whether or not breast cancer in Taiwan is influenced by factors established for high/moderate areas. The present case–control study was undertaken to investigate this subject.

*Received 21 May 1996*
*Revised 18 September 1996*
*Accepted 19 September 1996*

*Correspondence to*: C-Y Shen, Institute of Biomedical Sciences, Academia Sinica, Taipei, 11529, Taiwan

### SUBJECTS AND METHODS

### Case and control selection

This case–control study was conducted at Mackay Memorial Hospital, Taipei, Taiwan, from January 1993 to December 1994. On the basis of hospital chart number, the cases were 244 women randomly selected from subjects with newly diagnosed (incident) and pathologically confirmed breast carcinoma in the age range 20–80 years. The histopathological profile included 227 cases of infiltrating ductal carcinoma and 17 cases of intraductal or intralobular carcinoma. This sample of female patients constituted about 30% of all the women with breast cancer attending our breast cancer clinic during the study period.

To serve as comparable and representative controls, 450 (unmatched) women of the same age distribution were, on the basis of chart number, randomly recruited from patients attending ophthalmology outpatient clinic in the same hospital. The controls constituted about 20% of all women attending the ophthalmology clinic during the same study period as cases. Only a very low proportion (2%) of selected women (cases and controls) refused to participate in this study. On the basis of average family income and educational level, both case and control groups showed a high degree of homogeneity, and almost all (>90% of both cases and controls) represented a population of middle-class women in Taiwan.

### Structured questionnaire and standardized interview

After receiving informed consent, we used a structured questionnaire to collect detailed information on demographic, lifestyle and medical history data as well as details of family history of breast and other cancers. A complete menstrual and pregnancy history was obtained for all participants. We also obtained information regarding contraceptive behaviour and history of induced or spontaneous abortion. For each induced or spontaneous abortion reported, a detailed history of the event was obtained. More specifically, in

**Table 1** Distribution of risk factors and associated age-adjusted odds ratios (aORs) of breast cancer among patients and control subjects, Taiwan, 1993–94

| | All ($n$ = 694) | | | Premenopause ($n$ = 353) | | | Post-menopause ($n$ = 341) | | |
|---|---|---|---|---|---|---|---|---|---|
| **Risk factor** | **Case** | **Control** | **aOR (95% CI)** | **Case** | **Control** | **aOR (95% CI)** | **Case** | **Control** | **aOR (95% CI)** |
| Mother or sister with breast cancer | | | | | | | | | |
| No | 225 | 442 | 1.00 | 111 | 228 | 1.00 | 114 | 214 | 1.00 |
| Yes | 19 | 8 | 4.66 | 10 | 4 | 5.33 | 9 | 4 | 4.61 |
| | | | (2.07–11.4) | | | (1.71–20.0) | | | (1.36–18.4) |
| Previous breast biopsy or operation | | | | | | | | | |
| No | 211 | 427 | 1.00 | 96 | 219 | 1.00 | 115 | 208 | 1.00 |
| Yes | 33 | 23 | 2.87 | 25 | 13 | 4.24 | 8 | 10 | 1.32 |
| | | | (1.65–5.06) | | | (2.10–8.92) | | | (0.47–3.57) |
| Having a smoking history | | | | | | | | | |
| No | 231 | 440 | 1.00 | 113 | 225 | 1.00 | 118 | 215 | 1.00 |
| Yes | 13 | 10 | 2.42 | 8 | 7 | 2.68 | 5 | 3 | 3.57 |
| | | | (1.05–5.76) | | | (0.93–7.97) | | | (0.82–18.4) |
| Age at menarche (years) | | | | | | | | | |
| > 13 | 182 | 369 | 1.00 | 89 | 169 | 1.00 | 93 | 200 | 1.00 |
| 13 | 50 | 65 | 1.35 | 25 | 48 | 1.03 | 25 | 17 | 2.87 |
| <13 | 12 | 16 | 1.69 | 7 | 15 | 1.06 | 5 | 1 | 7.84 |
| | | | $P$=0.05* | | | $P$=0.89* | | | $P$<0.001* |
| Menstrual cycle history (years) | | | | | | | | | |
| <10 | 8 | 20 | 1.00 | 1 | 20 | 1.00 | 7 | 0 | 1.00 |
| 10–20 | 37 | 94 | 1.93 | 34 | 90 | 2.83 | 3 | 4 | – |
| >20 | 199 | 336 | 3.61 | 86 | 122 | 7.84 | 113 | 214 | – |
| | | | $P$=0.01* | | | $P$=0.003* | | | |
| Regular menstrual cycle | | | | | | | | | |
| No | 50 | 121 | 1.00 | 26 | 67 | 1.00 | 24 | 54 | 1.00 |
| Yes | 194 | 329 | 1.43 | 95 | 165 | 1.67 | 99 | 164 | 1.45 |
| | | | (1.00–2.09) | | | (1.00–2.87) | | | (0.83–2.60) |
| No. of full-term pregnancies | | | | | | | | | |
| >2 | 128 | 258 | 1.00 | 39 | 68 | 1.00 | 89 | 190 | 1.00 |
| 1–2 | 87 | 117 | 1.11 | 61 | 92 | 1.10 | 26 | 25 | 2.22 |
| 0 | 29 | 75 | 1.24 | 21 | 72 | 1.21 | 8 | 3 | 5.69 |
| | | | $P$=0.11* | | | $P$=0.87* | | | $P$=0.02* |
| Age at first full-term pregnancy (years) | | | | | | | | | |
| No pregnancy | 29 | 75 | 0.78 | 21 | 72 | 0.49 | 8 | 3 | 5.93 |
| ≥ 25 | 107 | 157 | 1.38 | 59 | 91 | 1.09 | 48 | 66 | 1.62 |
| <25 | 108 | 218 | 1.00 | 41 | 69 | 1.00 | 67 | 149 | 1.00 |
| | | | $P$=0.48* | | | $P$=0.32* | | | $P$=0.12* |
| Breast feeding | | | | | | | | | |
| No | 107 | 151 | 1.00 | 75 | 130 | 1.00 | 32 | 21 | 1.00 |
| Yes | 137 | 299 | 0.62 | 46 | 102 | 0.56 | 91 | 197 | 0.41 |
| | | | (0.42–0.89) | | | (0.34–0.91) | | | (0.22–0.78) |
| Use of oral contraceptive | | | | | | | | | |
| No | 179 | 353 | 1.00 | 80 | 172 | 1.00 | 99 | 181 | 1.00 |
| Yes | 65 | 97 | 1.30 | 41 | 60 | 1.30 | 24 | 37 | 0.68 |
| | | | (0.90–1.87) | | | (0.79–2.11) | | | (0.36–1.27) |
| Spontaneous abortion | | | | | | | | | |
| No | 191 | 358 | 1.00 | 95 | 196 | 1.00 | 96 | 162 | 1.00 |
| Yes | 53 | 92 | 1.11 | 26 | 36 | 1.34 | 27 | 56 | 0.88 |
| | | | (0.75–1.62) | | | (0.75–2.36) | | | (0.50–1.50) |
| Body mass index (BMI, kg m$^{-2}$) | | | | | | | | | |
| <25 | 172 | 358 | 1.00 | 110 | 206 | 1.00 | 62 | 152 | 1.00 |
| ≥ 25 | 72 | 92 | 1.21 | 11 | 26 | 0.84 | 61 | 66 | 1.34 |
| | | | (1.03–1.42) | | | (0.65–1.07) | | | (1.06–1.71) |

*Mantel chi-square test.

this study, regular menses was recorded as more than ten cycles of menses of regular length and interval within a 1-year period. The history of menstrual cycle represented the number of years exposed to menstrual cycles and was based on the age at menarche and ages at interview for premenopausal women and ages at the time of menarche and menopause for post-menopausal women. The number of children or full-term pregnancies reported was used to define the status of parous or nulliparous. Previous breast feeding was defined as having breast fed for more than 2 months. The weight/height ratio, used to calculate body mass index (BMI),

© Cancer Research Campaign 1997

**Table 2** Logistic regression analysis of multiple risk factors of breast cancer on women in Taiwan, 1993–94[a]

| | All women (*n* = 694) | | Premenopausal (*n* = 353) | | Post-menopausal (*n* = 341) | |
|---|---|---|---|---|---|---|
| **Risk factor** | **OR** | **95% CI** | **OR** | **95% CI** | **OR** | **95% CI** |
| Menopausal status | | | | | | |
| Post *vs* pre | 2.00 | 1.19–3.38 | | | | |
| Mother or sister with breast cancer | | | | | | |
| Yes *vs* no | 4.69 | 1.99–12.0 | 6.08 | 1.81–24.4 | 3.89 | 1.07–16.4 |
| Previous breast biopsy or operation | | | | | | |
| Yes *vs* no | 2.71 | 1.52–4.90 | 3.83 | 1.81–8.44 | 1.46 | 0.48–4.29 |
| Having a smoking history | | | | | | |
| Yes *vs* no | 2.68 | 1.11–6.67 | 3.28 | 1.01–10.9 | 4.92 | 0.93–30.9 |
| Menses history (years) | | | | | | |
| >20 *vs* 10–20 *vs* <10 | 2.25 | 1.46–3.57 | 3.35 | 1.60–7.24 | | |
| Age at menarche (years) | | | | | | |
| <13 *vs* 13 *vs* >13 | 1.20 | 0.87–1.65 | | | 2.67 | 1.43–5.23 |
| Regular menstrual cycle | | | | | | |
| Yes *vs* no | 1.50 | 1.01–2.24 | 1.77 | 1.01–3.17 | 1.35 | 0.74–2.50 |
| Breast feeding | | | | | | |
| Yes *vs* no | 0.57 | 0.38–0.85 | 0.56 | 0.32–0.99 | 0.48 | 0.24–0.98 |
| No. of full-term pregnancies | | | | | | |
| | 0.94 | 0.84–1.06 | 1.20 | 0.96–1.50 | 0.84 | 0.72–0.99 |
| Body mass index (BMI, kg $m^{-2}$) | | | | | | |
| ≥ 25 *vs* <25 | 1.16 | 0.98–1.39 | 0.80 | 0.61–1.04 | 1.48 | 1.14–1.94 |

[a]The following variables do not reach statistical significance ($P$=0.05): the age at first full-term pregnancy, ever use of oral contraceptives, having history of spontaneous or induced abortion.

represented the average weight/height within the past 4 years. The structured questionnaire was administered at the time of recruitment. All interviews were carried out by two experienced nurse interviewers who had been thoroughly familiarized with the study protocol.

This study did not institute 'blinding' procedures with respect to subjects' case status in the stages of data collection. Therefore, it was possible that women who were diagnosed with breast cancer were more likely to provide more detailed complete information about past exposure history than controls. However, the investigators and interviewers were fully informed about the possibility of recall/interviewer biases and their potential impact on our study. Multiple efforts, including standardization of wording in the interview and repeated interview for some same subjects (15% for both cases and controls), were made to evaluate consistency and to minimize such biases.

### Data analysis and statistical methods

Given that the same factors can have differing effects (and of differing magnitudes) on breast cancer risk during premenopausal or post-menopausal periods, we performed univariate analysis of suspected risk factors and calculated associated odds ratios of breast cancer for all women or for women grouped by pre/post-menopausal periods. Proportions for known or suspected risk factors for breast cancer were computed for the case patients and controls. Conventional cut-offs were used to classify or dichotomize some continuous variables, for example BMI. The significance of any difference in proportions was tested by the chi-square test, and the odds ratio (OR) and corresponding 95% confidence interval (CI) were estimated. The Mantel chi-square test for trends (Mantel, 1963) was used to examine the dose–response relationship for the breast cancer risk estimates of various categories of single risk factors. Logistic regression analyses based on all women or for women grouped by menopausal status were performed to estimate multivariate adjusted OR and 95% CI. To obtain a model with biological plausibility, we included all known risk factors in the logistic models regardless of their statistical significance. These known risk factors included the age of study participant and genetic predisposition for breast cancer (indicated by having a family history, i.e. mother or sisters had breast cancer) (Kelsey, 1993). A backward elimination procedure (Kleinbaum et al, 1982) was used to select the optimal model. To examine any possible interaction among risk factors and to compare their influence in pre/post-menopausal periods, we also retained any risk factors that had been shown to be significant in either premenopausal or post-menopausal models throughout all multivariate analyses. All $P$-values were two-tailed.

### RESULTS

Of 694 study participants, there were 353 premenopausal women (121 cases and 232 controls, among them 16 were perimenopausal) and 341 post-menopausal women (123 cases and 218 controls). Premenopausal cases were on average older than their controls (average age 38.8 *vs* 36.3 years), but post-menopausal cases were on average younger than their controls (average age 53.8 *vs* 59.1 years). Compared with premenopausal women, post-menopausal women demonstrated a slightly increased risk for breast cancer (OR 1.60, 95% CI 0.99–2.61) after adjusting the age of study participants. Among post-menopausal women, older age at menopause (defined as 45 years of age or older at menopause) was found to increase breast cancer risk (OR 1.45), but its effect was not statistically significant ($P$=0.28). Because this study was not based on a matched design and many risk factors were age related, univariate analyses of suspected risk factors were performed to calculate associated age-adjusted OR and CI of breast cancer (Table 1).

© Cancer Research Campaign 1997

First, we considered risk factors that might be related to genomic damage or genetic predisposition. As expected, having a family history of breast cancer in mother or sisters was highly associated with increased risk of breast cancer in both premenopausal or post-menopausal groups. A history of breast biopsy or operation also increased risk but, in post-menopausal women, this association was not significant. Smoking history was also found to correlate with breast cancer risk but, after stratification by menopausal status, smokers no longer displayed any elevated risk with statistical significance (Table 1).

Next, we considered risk factors related to menstrual cycle that might reflect the influence of reproductive hormones. For all women studied, breast cancer risk was related to younger age of menarche ($P$=0.05 for linear trend), longer history of menstrual cycle ($P$=0.01 for linear trend) and regular menstrual cycle ($P$=0.05). Individually, premenopausal breast cancer showed a closer link with a longer history of menses ($P$=0.003 for linear trend), whereas post-menopausal breast cancer was related to earlier age at menarche ($P$<0.001 for linear tread) (Table 1).

With regard to risk factors related to pregnancy, on the basis of a suggested influence of full-term pregnancy on breast cells (Russo et al, 1982), an increase in full-term pregnancies would be expected to correlate with a decreased risk of breast cancer in post-menopausal women (Table 1). However, in our premenopausal women, no notable difference in these protective effects was observed. Furthermore, the suggested protective effect of younger age at the first full-term pregnancy was not observed in our women regardless of their menopause status. In contrast, the protective effect of previous breast feeding on breast cancer was obvious (OR = 0.62) and was consistently observed in both pre/post-menopausal women (Table 1).

No association was found between the use of oral contraceptives (OC) and breast cancer risk in our study participants (Table 1). Furthermore, no association with increased risk could be established between the time of first use of OC, either at ages younger than 25 or before the first birth, the length of oral contraceptive use and the years since first use (data not shown). History of abortion, either spontaneous or induced, was not found to be correlated to breast cancer. Further examination was carried out to determine if any correlation could be established between cancer risk and the timing and/or frequency of abortion; no association was found (data not shown).

A positive correlation was found between a higher value of body mass index [BMI (kg $m^{-2}$), weight/(height)$^2$)] and post-menopausal breast cancer ($P$<0.05). In contrast, a slight negative correlation was found between premenopausal breast cancer and BMI ($P$>0.05) (Table 1).

Subsequent to the consideration of all factors in the univariate analysis, the results of the logistic regression analysis which simultaneously assesses multiple risk factors for premenopausal and post-menopausal breast cancers are shown in Table 2. These results are similar to those obtained by univariate analysis. Having a family history of breast cancer in mother or sister played by far the most important role in the correlation of risk to breast cancer. The multivariate adjusted OR was as high as 4.69 for those who had family history of breast cancer, and the magnitude was similar to that reported in previous studies (Kelsey, 1993). Reproductive hormones, indicated by longer years of menses in premenopausal women or younger age at menarche in post-menopausal women, significantly increased risk. Previous history of breast biopsy or operation, smoking and regular menstrual cycle were only associated with premenopausal breast cancer. While post-menopausal obesity appeared to increase risk, premenopausal obesity, in contrast, did not significantly decrease risk as suggested in previous studies (Hunter and Willett, 1993; Kelsey, 1993). The protective effect of breast feeding was statistically significant, with the OR as low as 0.5 for both premenopausal or post-menopausal women who had breast fed. Increased numbers of full-term pregnancies were correlated with decreased risk only in post-menopausal breast cancer. The effect of other risk factors, including the age at first full-term pregnancy, the use of OC and the history of either spontaneous or induced abortion, was not found to be significantly associated with breast cancer.

### DISCUSSION

The present study further characterizes breast cancer epidemiology, especially in determining the risk profiles related to a low-incidence area. Both cases and controls were chosen intentionally from the same hospital during the same study period. This design ensured that our controls were able to represent the background population from which the cases were derived. We selected our controls from women attending outpatient clinics of the ophthalmology department. These women were diagnosed as having conjunctivitis, cataract, retinal disease or glaucoma, and most, if not all, of the causes of these ophthalmic diseases or abnormalities are not related to reproductive factors or genetic predisposition to cancer.

As in Western countries, we found that a family history of breast cancer is an important factor contributing to breast cancer in Taiwan. This observed familial association is likely to imply genetic predisposition. Therefore, it is of interest to determine whether known breast cancer susceptibility genes, such as *BRCA1* (Mike et al, 1994) and *BRCA2* (Wooster et al, 1995), responsible for a proportion of breast cancers in Western countries, also play a role in breast cancer in low-incidence areas.

Cell division is considered to play a crucial role in the pathogenesis of cancer, and reproductive factors that increase mitotic activity in breast epithelium are presumed to increase risk (Pike et al, 1993). This may explain our finding that long history of menses in premenopausal women or early menarche in post-menopausal women increases breast cancer risk. In addition, women with irregular menses have less frequent ovulation, hence their exposure to progesterone which occurs only after ovulation is reduced (Spicer and Pike, 1995). Irregular cycles are, therefore, suggested to be associated with a lower breast cancer risk, as observed in our premenopausal breast cancer group. Overall, these findings indicate that although Asian women show an average 20 per cent reduction in oestradiol compared with Western women (Bernstein et al, 1990; Pike et al, 1993), breast cancer remains highly hormone dependent, as in high- to moderate-risk areas.

Experimental studies on full-term pregnancy in rats are shown to result in permanent differentiation in vulnerable breast stem cells, altering subsequent susceptibility to hormones (Russo et al, 1982). This suggests a decreased risk of breast cancer in women who have their first birth at a younger age or in women who have more full-term pregnancies. Our analyses, however, were consistent with such an effect only in part, with statistical significance observed only in post-menopausal women.

Our data provide evidence to support breast feeding as the most important protective factor. Certain mechanisms, including decreasing oestrogen production during lactation or flushing out of

© Cancer Research Campaign 1997

carcinogens, have been suggested to explain such an observation. This protective effect of lactation has been previously observed in other Chinese populations (Tao et al, 1988; Yuan et al, 1988), but is considered to be less obvious in Western populations. Traditional Taiwanese women tend to breast feed for a longer period because a higher proportion do not work outside the home, which explains why the protective effect was particularly marked in the Chinese population. Related to this protective effect could be the decreasing trend of breast feeding in Taiwan (90%, 1960s; 30%, 1980–90) which may be specifically correlated to the increasing incidence of Taiwanese breast cancer.

As in previous studies (Hunter and Willett, 1993), obesity during the post-menopausal years greatly increases breast cancer risk but has a slightly reduced risk during the premenopausal years. Endogenous oestrogen converted and released from adipose tissue after menopause is thought to be responsible for increased breast cancer risk in post-menopausal obese women (Kelsey, 1993).

It is widely recognized that breast cancer risk attained during the premenopausal period is not lost after menopause (Pike et al, 1993). Overall, the post-menopausal women in this study demonstrated a higher risk for breast cancer than premenopausal women, which is consistent with this trend.

Breast cancer incidence in native-born and USA-born Asian-Americans is approximately 50% and 75%, respectively, that of USA-born Whites and is approximately twice that of women residing in Asia (Ziegler et al, 1993; Hanley et al, 1995). Exposure to Western lifestyles is thought to have a substantial impact on the increased risk for breast cancer. These lifestyle-related factors may be operative by way of hormonal–reproductive mechanisms which promote onset of earlier menarche, later menopause or an increase in the proportion of nulliparity and post-menopausal obesity. Our findings suggest that breast cancer in Taiwan is similar with respect to hormonal–reproductive risk factors to that in high- to moderate-incidence areas. Similar hormonal–reproductive mechanisms suggested in migrant studies for the low incidence in Asian populations, may be important in explaining the low incidence or the increasing trend of breast cancer in Taiwan.

### REFERENCE

Bernstein L, Yuan JM, Ross RK, Pike MC, Hanisch R, Lobo R, Stanczyk F, Gao YT and Henderson BE (1990) Serum hormone levels in pre-menopausal Chinese women is Shanghai and white women in Los Angeles: results from two breast cancer case–control studies. *Cancer Causes Control* **1**: 51–58

Fisher B, Osborne CK, Margolese R and Bloomer W (1993) Neoplasms of the breast. In *Cancer Medicine*, 3rd edn, Holland JF, Frei E III, Bast RC, Kufe DW, Morton DL and Weichselbaum RR (eds), pp.1706–1774. Lea & Febiger: Philadelphia

Hanley AJ, Choi BC and Holowaty EJ (1995) Cancer mortality among Chinese migrants: a review. *Int J Epidemiol* **24**: 255–265

Hunter DJ and Willett WC (1993) Diet, body size and breast cancer. *Epidemiol Rev* **15**: 110–132

Kelsey JL (1993) Breast cancer epidemiology: summary and future directions. *Epidemiol Rev* **15**: 256–263

Kelsey JL, Gammon MD and John EM (1993) Reproductive factors and breast cancer. *Epidemiol Rev* **15**: 36–47

Kleinbaum DG, Kupper LL and Morgenstern H (1982) *Epidemiologic Research*. Van Nostrand Reinhold: New York

Mantel N (1963) Chi-square tests with one degree of freedom: extensions of the Mantel–Haenszel procedure. *J Am Stat Assoc* **58**: 690–700

Miki Y, Swensen J, Shattuck-Eidens D, Futreal PA, Harshman K, Tartigian S, Liu Q, Cochran C, Bennett LM, Ding W, Bell R, Rosenthal J, Hussey C, Tren T, McClure M, Frye C, Hattier T, Phelps R, Haugen-Strano A, Katcher H, Yakumo K, Gholami Z, Schaffer D, Stone S, Bayer S, Wray C, Bogden R, Dayanath P, Ward J, Tonin P, Narod S, Bristow PK, Norris FH, Helvering L, Morrison P, Rosteck P, Lai M, Barrett JC, Lewis C, Neuhausen S, Cannon-Albright L, Gololgar D, Wiseman R, Kamb A and Skolnick MH (1994) A strong candidate for the breast and ovarian cancer susceptibility gene *BRCA1*. *Science* **266**: 66–71

Parkin DM, Pisani P and Ferlay J (1993) Estimates of the worldwide incidence of eighteen major cancers in 1985. *Int J Cancer* **54**: 594–606

Pike MC, Henderson BE and Casagrande JT (1981) The epidemiology of breast cancer as it relates to menarche, pregnancy, and menopause. In *Hormones and Breast Cancer*, Pike MC, Siiteri PK and Welsch CW (eds), pp 3–18. Cold Spring Harbor Laboratory Press: Cold Spring Harbor NY

Pike MC, Spicer DV, Dahmoush L and Press MF (1993) Estrogens, progestogens, normal breast cell proliferation, and breast cancer risk. *Epidemiol Rev* **15**: 17–35

Rosenberg L, Metzger LS and Palmer JR (1993) Alcohol consumption and risk of breast cancer: a review of the epidemiologic evidence. *Epidemiol Rev* **15**: 133–144

Russo J, Tay LK and Russo IH (1982) Differentiation of the mammary gland and susceptibility to carcinogenesis. *Breast Cancer Res Treat* **2**: 5–73

Sattin RW, Rublin GL, Webster LA, Huezo CM, Ory HW, Wingo PA and Layde PM (1985) Family history and the risk of breast cancer. *JAMA* **253**: 1908–1913

Spicer DV and Pike MC (1995) Hormonal manipulation to prevent breast cancer. *Science and Medicine* **2**: 58–67.

Tao SC, Yu MC, Ross RK, and Xiu KW (1988) Risk factor for breast cancer in Chinese women of Beijing. *Int J Cancer* **42**: 495–498

Trichopoulos D, Macmahon B and Cole P (1972) The menopause and breast cancer risk. *J Natl Cancer Inst* **48**: 605–613

Wooster R, Bignell G, Lancaster J, Swift S, Seal S, Mangion J, Collins N, Gregory S, Gumbs C, Micklem G, Barfoot R, Hamoudi R, Patel S, Rice C, Biggs P, Hashim Y, Smith A, Connor F, Arason A, Gudmundsson J, Ficenec D, Kelsell D, Ford D, Tonin P, Bishop DT, Spurr NK, Ponder BAJ, Eeles R, Peto J, Devilee P, Cornelisse C, Lynch H, Ncord S, Lenoir G, Egilsson V, Barkadottir RB, Easton DF, Bentley DR, Futreal PA, Ashworth A and Stratton MR (1995) Identification of the breast cancer susceptibility gene *BRCA2*. *Nature* **378**: 789–792

Yuan JM, Yu MC, Ross RK, Gao YT, and Henderson BE (1988) Risk factors for breast cancer in Chinese women in Shanghai. *Cancer Res* **48**: 1949–1953

Ziegler RG, Hoover RN, Pike MC, Hildesheim A, Nomura AMY, West DW, Wu-Williams AH, Kolonel LN, Horn-Ross PL, Rosenthal JF and Hyer MB (1993) Migration patterns and breast cancer risk in Asian-American women. *J Natl Cancer Inst* **85**: 1819–1827

© Cancer Research Campaign 1997